# Reanalysis data fusion-based tropical cyclone formation prediction network

**DOI:** 10.1038/s41598-026-58904-1

**Published:** 2026-06-23

**Authors:** Ying Hai, Yuan Peng, Yuchen Wang, Zhiyong Cheng

**Affiliations:** 1 Chongqing Sub-bureau of Southwest China Regional Air Traffic Management Bureau CAAC, Chongqing, 401120 China; 2 CETC Taiji Computer Co., Ltd, Beijing, China; 3 Harbin Engineering University, Harbin, China; 4 CETC Big Data Research Institute, Guizhou, China; 5 Chaohu University, Hefei, China

**Keywords:** Reanalysis data, Tropical cyclone formation prediction, Data fusion, Deep learning, Computational biology and bioinformatics, Mathematics and computing

## Abstract

This paper proposes a tropical cyclone formation prediction network based on pyramid attention feature extraction and multi-scale feature fusion, aiming to enhance the prediction of whether tropical cloud cluster (TCC) precursors intensify to tropical storm (TS) strength by integrating multi-source reanalysis data. First, reanalysis data from ERA5 and NCEP/NCAR are represented as images at different scales and labeled according to tropical cyclone (TC) formation events derived from the TCC and IBTrACS datasets. The labels include information on whether TC formation occurred and its location. Then, a feature extraction module based on a Pyramid Attention Mechanism (PAM) is designed to extract features related to TC formation. Next, the features at different scales are input into a PAM-based feature fusion module that dynamically generates weights for different features to perform weighted fusion and unify the feature scales. Finally, a lightweight Convolutional Neural Network (CNN) is designed as the prediction module to predict TC formation occurrence and location. Experimental results over five independent data splits show that at a lead time of 24 hours, the proposed method achieves a Probability of Detection (POD) of *0.865 ± 0.012*, a False Alarm Ratio (FAR) of *0.326 ± 0.016*, and a location prediction Root Mean Square Error (RMSE) of *0.795 ± 0.033* grid units (*≈*437 km), demonstrating competitive performance in balancing POD and FAR. Ablation studies confirm the contribution of each proposed module to overall performance.

## Introduction

Tropical cyclones (TCs) refer to intense cyclonic storm systems that form over tropical or subtropical oceans, typically accompanied by strong winds and heavy rainfall. In recent years, with the intensification of global climate change, the frequency and intensity of TCs have been continuously changing, posing severe challenges to the economic and social development of coastal areas. Therefore, accurately predicting tropical cyclogenesis (TCG) is of great significance in mitigating the damage.

In traditional studies, such as in works of Kozar^1^, Pu^2^ and Dvorak^3^, the TCG prediction relies on numerical weather prediction (NWP) models, which simulate the physical processes of the atmosphere, such as the partial differential equations governing atmospheric dynamics, to forecast the formation and development of storms. However, due to the non-linear characteristics of the TCG process, NWP models face challenges such as data scarcity and model errors in predicting TCG. For example, Velden et al.^4^ proposed a Dvorak technique based on satellite imagery to estimate the intensity and location of TCs, but this method does not consider meteorological variables such as atmospheric pressure, leading to limited accuracy in predicting storm genesis. Liang et al.^5^ evaluated the TCG prediction performance of the European Center for Medium-Range Weather Forecasts (ECMWF), and their results indicated that the prediction accuracy is significantly related to the lead time. Approximately 60% of correct samples were accurately predicted within 24 hours, while the accuracy dropped to below 50% within 48 hours. Their results also indicated that NWP models struggle to determine whether a tropical disturbance will develop into a TC or decay into a low-pressure system. Physically, TC formation requires a favorable combination of thermodynamic and dynamic conditions, including sufficiently warm sea surface temperature, enhanced low-level cyclonic vorticity, adequate mid-level moisture, weak vertical wind shear, and a supportive large-scale synoptic environment. Diagnostic studies of tropical cloud clusters show that these ingredients are necessary but not sufficient: many cloud clusters exhibit some favorable conditions yet fail to organize into tropical storms because convective organization, environmental shear, dry-air intrusion, and synoptic-scale forcing evolve stochastically^6,7^. This intrinsic uncertainty is also reflected in verification studies of numerical model genesis forecasts, where forecast skill depends strongly on basin, lead time, disturbance definition, and verification protocol^5,8,9^.

Benefiting from the successful applications of deep learning in fields such as image recognition and natural language processing, researchers, such as in works of Park^10^, Zhang.T^11^, Zhang.R^12^ and Wang^13^, have attempted to utilize neural networks to capture the complex patterns and non-linear relationships in the TCG process. Neural network models such as Convolutional Neural Networks (CNNs)^14^, Recurrent Neural Networks (RNNs)^15^, and Long Short-Term Memory (LSTM)^16^ networks can simultaneously process large-scale meteorological data and satellite imagery, extracting useful features to improve prediction accuracy. Compared to traditional models, neural network models reduce reliance on physical processes and focus more on data-driven pattern recognition. To enhance the prediction performance of TCG, researchers often integrate multiple data sources, such as meteorological variables, satellite imagery, and historical storm data. Earlier statistical and machine-learning studies used objective TCC tracking, ocean-surface wind patterns, mesoscale convective systems, or reanalysis predictors to identify developing cloud clusters^10,11,13,17,18^. For instance, in Zhang’s work^12^, a CNN-based model was proposed to predict TCG by combining satellite images and ERA5 reanalysis data. The selected data were then combined with satellite data as layers input to the CNN model, with a fully connected layer outputting the prediction results. Mu et al.^19^ proposed a Swin-Transformer-based model that utilizes convolutional operators and self-attention mechanisms to capture the spatiotemporal features in the TCG process. They trained the model using satellite data and ERA5 analysis data, achieving a TCG prediction accuracy of 97.9% while reducing the false alarm ratio to 2.2%. However, the data used in the above models were manually selected after experiments, based on the principle that there is a certain correlation between the data. And the complexity and diversity of the TCG process may lead to manually selected data failing to comprehensively capture all relevant features, limiting the model’s prediction performance in certain situations. Furthermore, the above neural network models simply concatenate data from different sources, neglecting the potential relationships and interactions between different data sources. Therefore, how to effectively integrate multi-source data and extract semantic features related to TCG remains an urgent problem to be solved.

This paper proposes a TCG prediction method based on image fusion, aiming to enhance TCG prediction performance by integrating multi-source data. Specifically, this paper first represents data from different sources as images, then utilizes image fusion techniques to combine these images into a comprehensive image, and finally inputs the fused image into a neural network model for prediction. In traditional image processing, multiple features need to be extracted from the original images for fusion, such as edges, textures, and colors. In the context of meteorological data images, these features can represent the variations and spatial distributions of different meteorological variables across latitude and longitude. The design of traditional decomposition methods and fusion rules is time-consuming and complex, requiring the same representation and decomposition methods for different images. However, for meteorological data, the imaging methods of the source images are entirely different, making traditional fusion methods inapplicable.

Therefore, we employ neural networks to design an end-to-end image fusion method that can automatically learn the correlations between different data sources and extract features related to TCG. The model design mainly consists of three modules: a feature extraction module based on a Pyramid Attention Mechanism (PAM), a feature fusion module based on an improved PAM , and a prediction module based on a lightweight Convolutional Neural Network (CNN). Firstly, the feature extraction module employs a PAM to extract multi-scale features from data of different sources. Subsequently, the feature fusion module also utilizes the PAM strategy to fuse the extracted multi-scale features, where PAM dynamically generates weights for different features to perform weighted fusion of data from different sources, thereby enhancing features related to TCG. Then, the extracted features are used to train a neural network model to predict TCG. The proposed method in this paper is referred to as the Meteorological Data Fusion-based TCG Prediction Network (MDF-TNet), which has significantly fewer parameters than Transformer-based models. Experimental results demonstrate that the proposed method detects 85.9% of TC formation events while reducing the false alarm ratio to 32.0%. Moreover, MDF-TNet does not require manual data selection and can incorporate additional data sources as needed to enhance model performance.

The main contributions of this paper are summarized as follows:

1. A novel end-to-end multi-source reanalysis data fusion strategy is proposed for TC formation prediction, which automatically learns the correlations between ERA5 and NCEP/NCAR data without requiring manual variable selection.
2. A PAM-based feature extraction and fusion architecture is designed that dynamically generates attention weights to fuse multi-scale features from heterogeneous data sources, achieving effective fusion with a lightweight model.
3. The model jointly predicts both the occurrence and the spatial location of TC formation events through a combined category-position loss function, a capability that has rarely been explored in existing TC formation prediction studies.
4. Comprehensive ablation studies are conducted to validate the contribution of each proposed module, including data source fusion, the PAM mechanism, and the position loss component.

## Data

The data we use mainly comes from five sources: the European Centre for Medium-Range Weather Forecasting Re-Analysis(ERA5)^20,21^; TCC data^6^ from the National Hurricane Center (NHC); the global meteorological reanalysis dataset provided by the National Centers for Environmental Prediction/National Centers for Atmospheric Research (NCEP/NCAR); and the International Best Track Archive for Climate Stewardship (IBTrACS)^22^. The following is a detailed introduction to the dataset usage.

### ERA5 reanalysis data

ERA5 provides a total of 37 atmospheric layers from 1000 to 1 hPa, as well as data in the vertical direction over ocean and land surfaces. The data resolution for ocean variables is 0.25 degrees. ERA5 is a global meteorological reanalysis dataset provided by the European Centre for Medium-Range Weather Forecasting (ECMWF), covering the period from 1979 to the present. The ERA5 dataset offers high-resolution meteorological variables, including temperature, humidity, wind speed, and atmospheric pressure, with a temporal resolution of 6 hours and a spatial resolution of 0.25 degrees. In this paper, we selected the following variables from the ERA5 dataset as input features for the model: Sea Surface Temperature (SST), Mean Sea Level Pressure (MSLP), Relative Vorticity (Vo), Relative Humidity (RH), U wind component (U and U10), and V wind component (V and V10) at 0 and 10 meters above the surface. To better understand the spatial structure of the data, we selected data from eight pressure levels ranging from 850 hPa to 200 hPa, specifically 850 hPa, 800 hPa, 700 hPa, 600 hPa, 500 hPa, 400 hPa, 300 hPa, and 200 hPa. Since neural networks are sensitive to scalar data, we further converted U, V, U10, and V10 into Wind Speed (WS and WS10) and Wind Direction (WD and WD10). The formulas for converting the vector components of wind into scalar speed and direction are shown in Eq. (1), which is similar as in Zhang’s work^12^.

1 $$\begin{aligned} \begin{aligned} WS&= \sqrt{U^2 + V^2},\\ WD&= \left\{ \begin{array}{ll} \textrm{arctan}(\frac{U}{V}) \times \frac{180}{\pi } + 180, & V>0 \\ \textrm{arctan}(\frac{U}{V}) \times \frac{180}{\pi } + 0, & U<0\& ~V<0 \\ \textrm{arctan}(\frac{U}{V}) \times \frac{180}{\pi } + 360, & U>0\& ~V<0 \\ \end{array} \right. \end{aligned} \end{aligned}$$

where $\textrm{arctan}()$ is the arctangent function.

### NCEP/NCAR reanalysis data

NCEP/NCAR is a global meteorological reanalysis dataset provided by the National Centers for Environmental Prediction/National Centers for Atmospheric Research (NCEP/NCAR), covering the period from 1948 to the present. The NCEP/NCAR dataset offers various meteorological variables, including temperature, humidity, wind speed, and atmospheric pressure. In this paper, we selected the NCEP FNL Operational Model Global Tropospheric Analyses data (d083002) from the NCEP/NCAR dataset as the base data. This data includes multiple meteorological variables, with data starting from July 30, 1999, to the present, a temporal resolution of 6 hours, and a spatial resolution of 1 degree. We selected the Sea Level Pressure (SLP), 500 hPa Geopotential Height (HGT), 700 hPa Relative Humidity (RHUM), 850 hPa Relative Vorticity (VOR), Vertical Wind Velocity/Speed (VS) and Wind Field (UV) as input features. Similar to the ERA5 data, we also used Eq. (1) to convert U and V into WS850, and WD850.

### TCC data

The Global Tropical Cloud Cluster (TCC) dataset was created by Hennon et al.^6^, based on infrared cloud-top temperature (IR TBB) and visible (VIS) imagery from satellite data, covering the period from 1980 to the present. The dataset provides information on TCC time, latitude, longitude, and lead time (LT), with a temporal resolution of 3 hours. TCCs that develop into TCs are labeled as “dev,” while those that do not develop into TCs are labeled as “non-dev.” In this paper, TCC data is used as label data to indicate whether a TCC precursor intensifies to tropical storm (TS) strength, while TCCs that did not develop serve as negative samples.

### IBTrACS best track data

The International Best Track Archive for Climate Stewardship (IBTrACS)^22^ is a tropical cyclone best track dataset created jointly by multiple meteorological agencies, covering the period from 1842 to the present. The data from 1842 to 1978 is historical data, while the data from 1979 to the present is real-time data. Therefore, data after 1979 can be matched with ERA5 and NCEP/NCAR reanalysis data. Since the TCC dataset does not have clear labels for data after 2010, we selected TC formation data from the IBTrACS dataset to match with the reanalysis data. The matched data serves as labels to indicate the time and location of TC formation events.

#### Note on task definition

It should be noted that the labeling strategy differs between the two sub-periods. For 1982–2009, the “dev” and “non-dev” labels in the TCC dataset directly indicate whether a TCC precursor developed into a TC. For 2010–2022, since the TCC labels are incomplete, positive samples are supplemented using IBTrACS records where the maximum sustained wind speed exceeds 17 m/s (35 kt), which corresponds to the tropical storm threshold. Therefore, the prediction task in this paper is more precisely defined as predicting whether a TCC-identified precursor will intensify to at least TS strength, rather than strict cyclogenesis prediction for arbitrary disturbances. We clarify this definition throughout the revised manuscript.

All data and their sources are shown in Table 1.

**Table 1:** Data and Their Sources.

Variables	Abbreviation	DataSet
Sea Surface Temperature	SST	ERA5
Mean Sea Level Pressure	MSLP	ERA5
Relative Vorticity	Vo	ERA5
Relative Humidity	RH	ERA5
Wind Speed	WS	ERA5
Wind Direction	WD	ERA5
Wind Speed at 10m	WS10	ERA5
Wind Direction at 10m	WD10	ERA5
500 hPa Geopotential Height	HGT	NCEP/NCAR
700 hPa Relative Humidity	RHUM	NCEP/NCAR
850 hPa Relative Vorticity	VOR	NCEP/NCAR
850 hPa Wind Speed	WS850	NCEP/NCAR
850 hPa Wind Direction	WD850	NCEP/NCAR
Vertical Wind Velocity/Speed	VS	NCEP/NCAR

## Methodology

### Data preprocessing

According to the discussion in Wood’s work^7^, the causes of TCG vary in different regions. Therefore, selecting data from a global scale may introduce additional variables, leading to overall model inaccuracies. Especially since the data we use are all human-understandable analysis data, each representing a physical quantity, and the information contained in different physical quantities may vary with latitude and longitude. Thus, we selected the Western North Pacific (WNP) region as the study area, ranging from $0^{\circ }$ to $30^{\circ }$N latitude and $100^{\circ }$ to $180^{\circ }$E longitude. The reasons are listed as: 1. The WNP is one of the most active regions for tropical storms globally, generating more than 36% of the world’s total tropical storms annually. 2. The countries near the WNP have large populations and developed economies, making TCs highly destructive to the economy and society. 3. The WNP region has a distinct TC seasonality, which can better capture the spatiotemporal characteristics of TCG. Therefore, we filtered the data from the WNP region in the above datasets.

Then a list of time periods containing TCG events is constructed. The portion from 1982 to 2009 in this list was determined based on the “dev” labels in the TCC dataset. For data labeled as “dev”, we recorded TCG time points where the wind speed exceeded 17 m/s, approximating these time points to the nearest 6 hour intervals. The 6 hour intervals refer to 00:00, 06:00, 12:00, and 18:00 UTC. The reason for this is to align the 3 hour temporal resolution of the TCC dataset with the 6 hour temporal resolution of the ERA5 and NCEP/NCAR datasets. For example, if a TCG event occurred at 9:00 UTC on August 15, 2010, we would approximate it to 12:00 UTC on August 15, 2010. For the portion from 2010 to 2022, since the TCC dataset was not well-labeled, we referred to Hennon’s work^6^ and used TCG events from the IBTrACS dataset to supplement the TCC dataset in constructing the time period list. The TCG events in the IBTrACS dataset refer to time points where the wind speed in the USA_WIND label exceeds $17\,\mathrm {m/s}$ (35 kts). TC events in the IBTrACS dataset that were close in time and location to events in the TCC dataset were considered as “dev” labels, and we used the same method as above to approximate them to the nearest 6 hour intervals.

We then constructed a list of negative sample time periods. The portion from 1982 to 2009 in this list was determined based on the “non-dev” labels in the TCC dataset. For data labeled as “non-dev”, we randomly sampled approximately twice the number of 6 hour intervals as the “dev” labels. For the portion from 2010 to 2022, since the TCC dataset was not well-labeled, we randomly sampled 6 hour intervals that existed in the TCC dataset but not in the IBTrACS dataset. Thus, the negative samples after 2010 are non-developing TCC candidates rather than arbitrary oceanic non-TC times. We retained the same approximate positive-to-negative ratio of 1:2 used for the 1982–2009 TCC labels when constructing the 2010–2022 samples, so that the classifier was trained and evaluated under a controlled class balance. In an operational environment, the actual ratio of non-developing disturbances to developing TCs can be substantially larger; therefore, the reported FAR should be interpreted as the false-alarm behavior under the sampled TCC-candidate dataset, not as the unconditional basin-wide false alarm frequency.

After constructing the lists of positive and negative sample events, we extracted the corresponding meteorological variables from the ERA5 and NCEP/NCAR datasets based on these time points. For each time point, we extracted meteorological variable data from 24 hours and 48 hours prior. For example, if a TCG event occurred at 12:00 UTC on August 15, 2010, we extracted meteorological variable data from 12:00 UTC on August 14, 2010, and 12:00 UTC on August 13, 2010. We then standardized the extracted meteorological variables using the Min-Max normalization method to maintain the relative relationships between the data. Importantly, the Min-Max normalization statistics (minimum and maximum values) were computed exclusively from the training set and then applied to the validation and test sets, preventing information leakage. For the small number of missing values in ERA5 and NCEP/NCAR data (primarily at coastal grid points), we performed bilinear interpolation from neighboring valid grid points before normalization. We then converted each data point into a multi-channel image according to the data format in Table 1. Among them, the Vo, RH, WS, and WD data from the ERA5 dataset are 4-channel images (one per pressure level group), while the other data are single-channel images. The images generated from the ERA5 dataset are *120 × 320* pixels, while those from the NCEP/NCAR dataset are *30 × 80* pixels, which serve directly as the network input dimensions.

We constructed the label set based on the time and location of TCG events from the IBTrACS dataset. The label set has a resolution of 5 degrees, covering the range from $0^{\circ }$ to $30^{\circ }$N latitude and $100^{\circ }$ to $180^{\circ }$E longitude. For each TCG event, we marked the 5-degree grid cell containing its latitude and longitude as 1, indicating that a TCG event occurred within that cell. For 5-degree grid cells where no TCG event occurred, we marked them as 0, indicating that no TCG event occurred within that cell. The constructed result is a 6x16 two-dimensional matrix.

Finally, we combined all the positive and negative sample data with the labels to form the final dataset. Each label corresponds to two sample data points, representing the meteorological variable data from 24 hours and 48 hours prior. We then split the dataset into training, validation, and test sets in an 8:1:1 ratio. The split was performed after grouping samples by disturbance/event, so that the 24 hour and 48 hour samples generated from the same event and samples from the same tropical disturbance (within 72 hours and 500 km) were assigned to the same subset. This procedure prevents the same disturbance from appearing simultaneously in the training, validation, and test sets. We further validated robustness through five independent disturbance-aware random splits, as reported in “Statistical Reliability" sect.” The final sample counts are: 990 positive samples (TC formation events) and 1980 negative samples (non-formation events). The training set contains 792 positive samples and 1584 negative samples, the validation set contains 99 positive samples and 198 negative samples, and the test set contains 99 positive samples and 198 negative samples.

### Reproducibility details

The complete preprocessing pipeline consists of the following steps: (1) Extract meteorological variables from ERA5 ($0.25^{\circ }$ resolution) and NCEP/NCAR FNL ($1^{\circ }$ resolution) for the WNP region ($0^{\circ }$–$30^{\circ }$N, $100^{\circ }$–$180^{\circ }$E) at each event time point. (2) Fill missing values via bilinear interpolation from neighboring grid points. (3) Convert wind vector components (U, V) to scalar wind speed and direction using Eq. (1). (4) Arrange each variable as a spatial image; multi-level variables (Vo, RH, WS, WD at 8 pressure levels) form 4-channel images. (5) Apply Min-Max normalization using statistics computed from the training set only. (6) Construct labels as *6 × 16* binary matrices based on IBTrACS TC formation locations at $5^\circ$ grid resolution.

The coordinate reference system is WGS84 with a regular latitude-longitude grid. All data processing was implemented in Python 3.9 using NumPy 1.24, xarray 2023.1, and scipy 1.10 for interpolation. The model was implemented in PyTorch 1.13.

### Model architecture

**Fig. 1:**
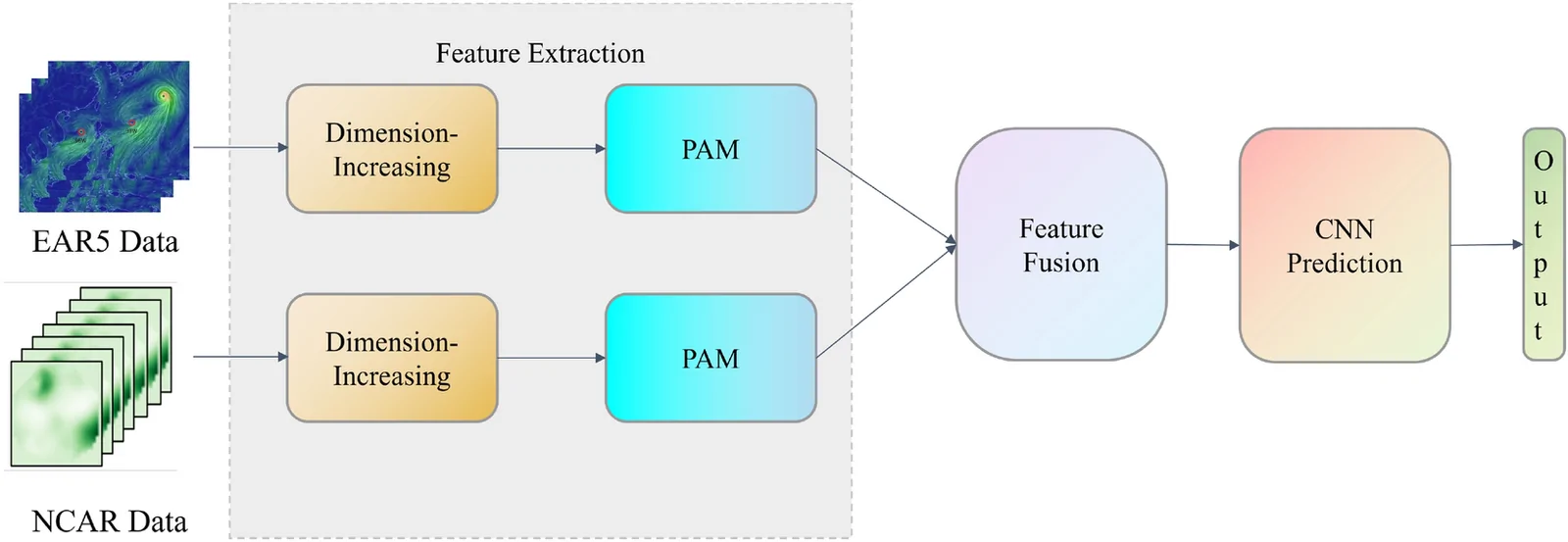
The overall architecture of MDF-TNet.

As described in the data preprocessing method, we obtained meteorological variable data from different sources and converted them into multi-channel images. These images contain spatial distribution information of different physical quantities across latitude and longitude. However, these images vary in size and represent different physical meanings, with most of the relationships between these physical meanings being unknown. Therefore, effectively fusing the image features is crucial in designing the model. To address this, we designed a TCG prediction network based on image fusion, referred to as MDF-TNet. The model mainly consists of three modules: a feature extraction module based on PAM, an improved feature fusion module based on PAM, and a prediction module based on a lightweight CNN. Figure 1 shows the overall architecture of MDF-TNet. Table 2 summarizes the detailed configuration of each module.

#### Feature extraction module

**Fig. 2:**
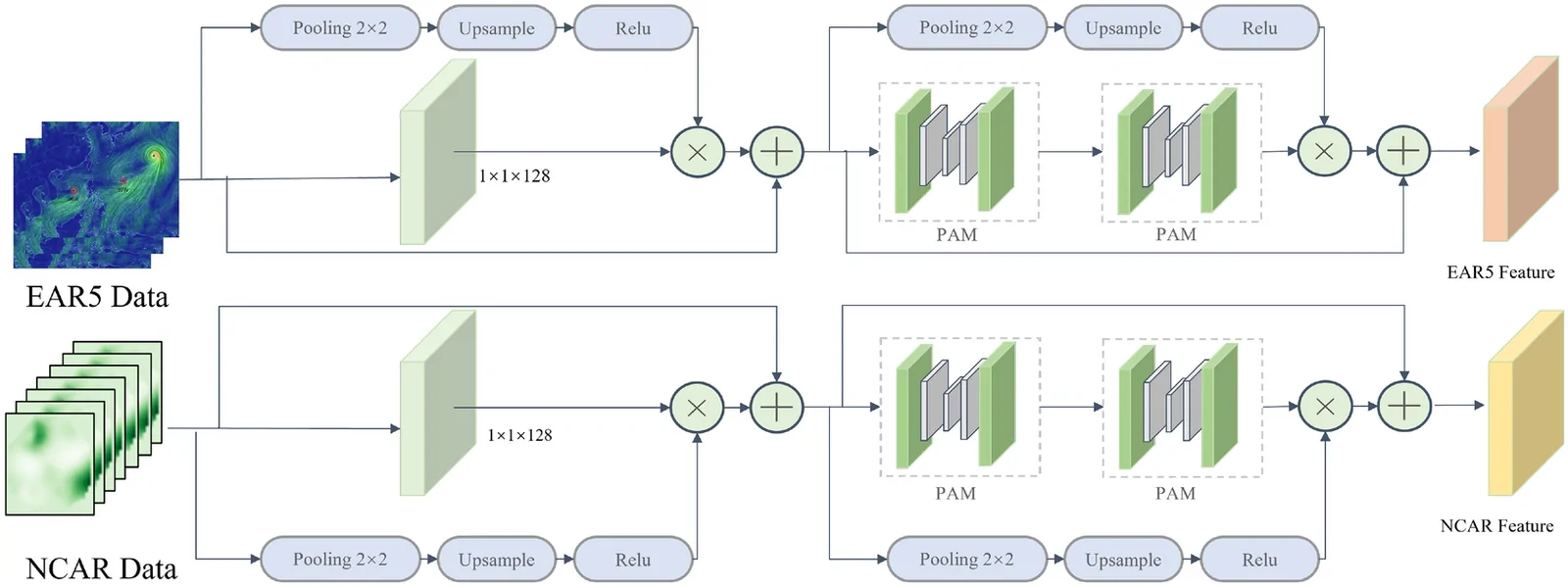
The architecture of Feature Extraction Module.

The feature extraction module mainly consists of two parts, as shown in Fig. 2. The first part is the dimension enhancement module, which uses skip connections combined with 1x1 convolutional kernels to increase the number of channels in the input images. Specifically, assuming the input image has a size of 120*×*320*×*1, where 120 is the height of the image, 320 is the width of the image, and 1 is the number of channels in the image. The dimension enhancement module first uses a 1x1 convolutional kernel to increase the number of channels of the input image to 127, then concatenates the input image with the enhanced image through a skip connection, resulting in an image of size 120*×*320*×*128. The purpose of this module is to increase the number of channels in the image thereby enhancing the expressive capability of the subsequent feature extraction module.

**Fig. 3:**
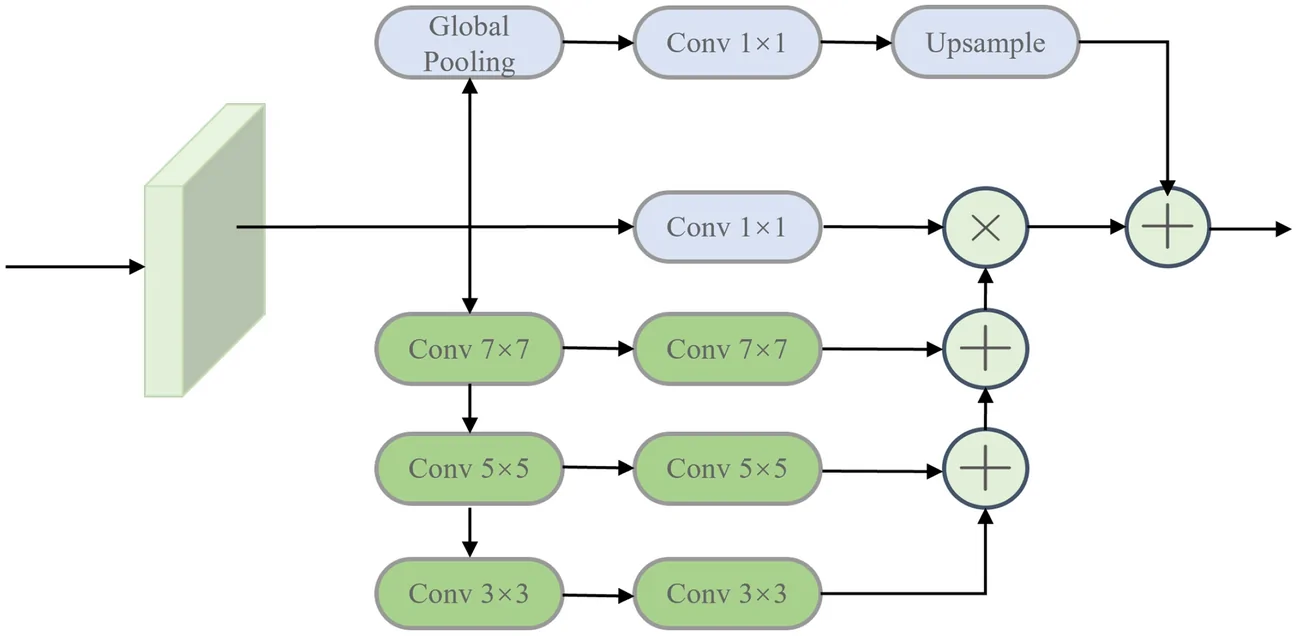
The architecture of Pyramid Attention Module.

The second part is the PAM module^23^. The PAM module is an improvement over the traditional feature pyramid, incorporating a mask module. The mask module uses an attention mechanism to weight the output features, providing multi-scale feature representations. Its mathematical expression is shown in Eq. (2).

2 $$\begin{aligned} \begin{aligned} F_{PAM} = (1+P_1(P_2(P_3(x))))\odot V(x) \end{aligned} \end{aligned}$$

where $P_1$, $P_2$, and $P_3$ represent convolutional layers with different kernel sizes, *V*(*x*) represents a convolutional operator, and *x* is the original feature. The operator *⊙* denotes element-wise multiplication between the attention mask and the value feature map. Padding, pooling, and upsampling are used inside the PAM branch so that $P_1(P_2(P_3(x)))$ and *V*(*x*) have the same spatial resolution; when channel numbers differ, a *1× 1* convolution is used for channel alignment before multiplication. The structure of the PAM module is shown in Fig. 3.

#### Feature fusion module

**Fig. 4:**
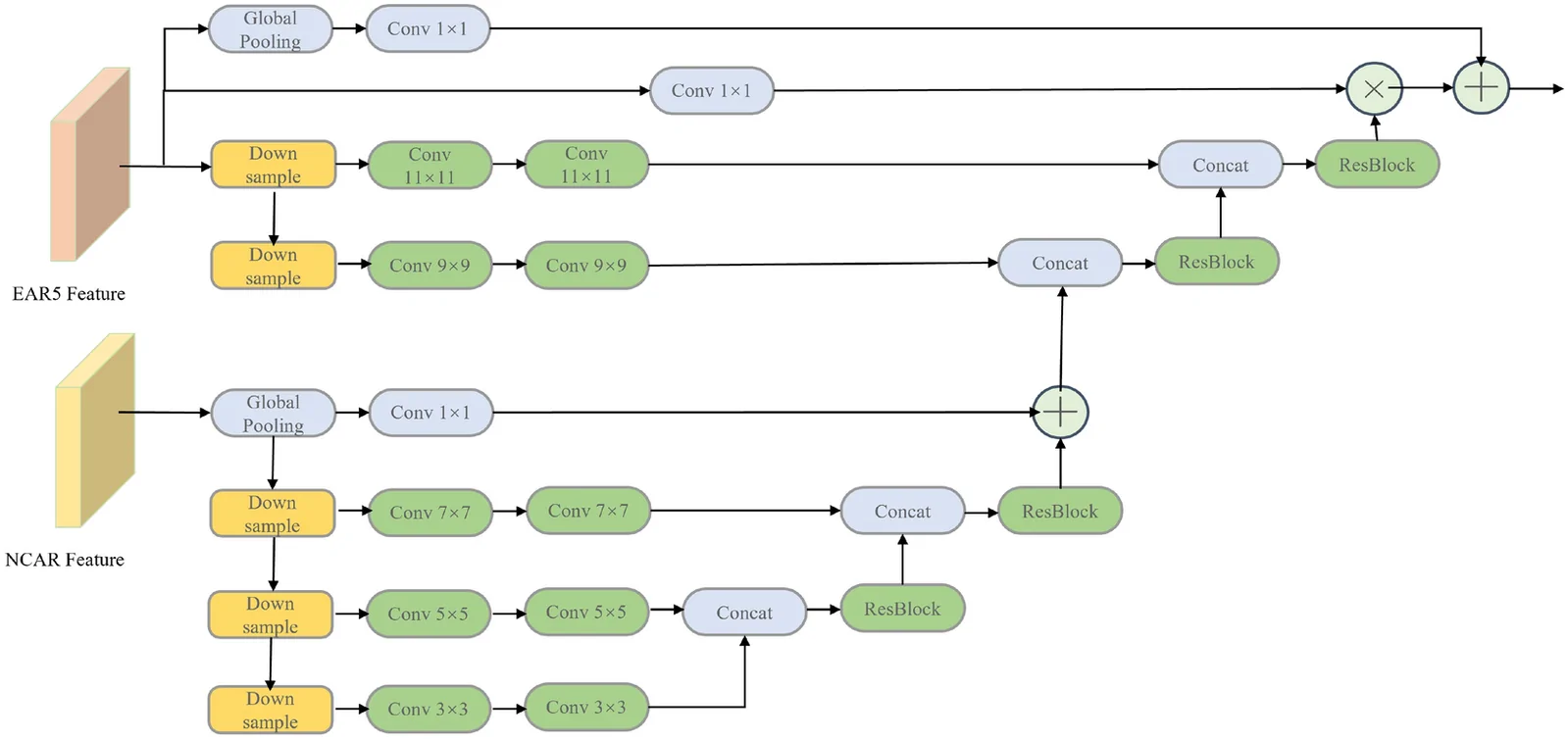
The architecture of Feature Fusion Module.

The purpose of the feature fusion module is to fuse features from different scales and sources. As shown in Table 1, we extracted data for 8 variables from the ERA5 dataset and data for 6 variables from one pressure level of the NCEP/NCAR dataset. Therefore, we extracted a total of 8x4+4+6x1=44 feature maps. These feature maps have sizes of 120x320 and 30x80, respectively. Thus, effectively fusing these feature maps of different sizes is crucial in designing the feature fusion module. A straightforward approach would be to downsample the 120x320 feature maps to 30x80, then concatenate all the feature maps along the channel dimension to obtain a unified feature map of size 30x80. However, this approach does not capture the correlations between different features and cannot distinguish the impact of different features on TCG. Therefore, we designed a feature fusion module based on PAM. The structure of this module is shown in Fig. 4.

The module first downsamples the 30*×*80 NCAR data feature maps through the PAM, then extracts features using two 7x7 convolutional kernels. Subsequently, the data continues to be downsampled and processed with smaller convolutional kernels to extract features at different scales. The purpose of downsampling is to ensure that the dimensions of the feature maps are similar, thereby enhancing the effectiveness of feature fusion. Then, the feature maps at different scales are concatenated instead of added, preserving the independence of the features.

All the obtained feature maps are summed with skip connections to obtain the fused features of the NCAR data. The processing of the ERA5 data is similar to that of the NCAR data, except that the initial size of the ERA5 data is 120x320. Therefore, the ERA5 data needs to be downsampled twice to match the size of the NCAR data. The feature maps of the NCAR data are concatenated with the ERA5 data after two downsamplings to obtain mixed features. The mixed features are further upsampled and concatenated in a pyramid manner, then weighted and summed with the original feature maps of the ERA5 data to obtain the final fused features. The upsampling is implemented through residual blocks. The final feature map has a dimension of 120x320.

In the proposed method, we fuse features of smaller scales with those of larger scales through upsampling and concatenation. The advantage of this approach is that it preserves more of the original feature information. We also use residual blocks^24^ to weight features at different scales. This residual blocks captures the relationships between different features. Additionally, the residual blocks can balance the relationship between the size and channels of the feature maps. Therefore, through the FFM module, we achieve the fusion of features at different scales while maximizing the preservation of original features and considering the relationships between different features.

#### Prediction module

**Fig. 5:**
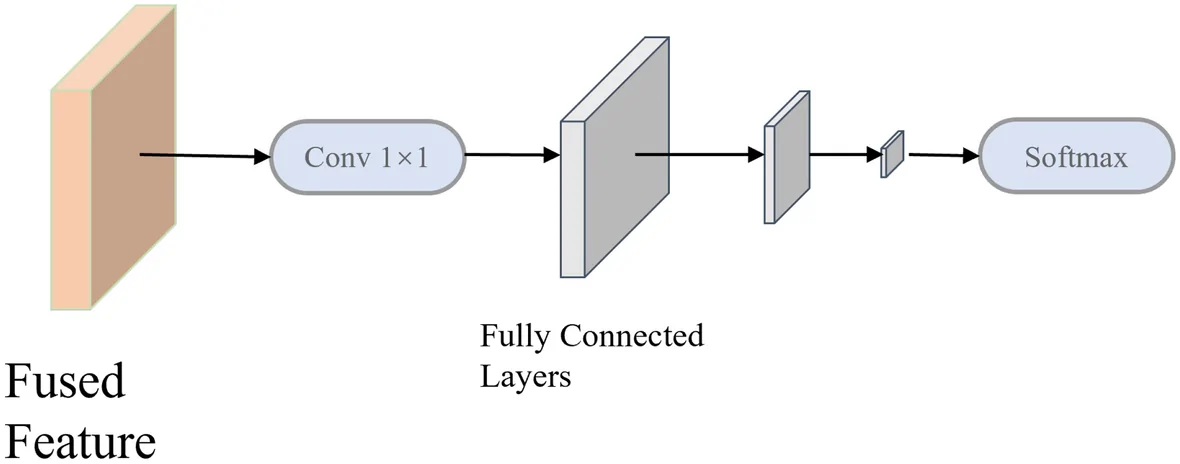
The architecture of Prediction Module.

The prediction module has two main purposes. The first purpose is to reduce the dimensionality of the fused feature map, resulting in a feature map of size 6x16. The second purpose is to achieve the final prediction. To this end, we designed a lightweight CNN as the prediction module. The structure of this module is shown in Fig. 5.

The module first uses a 1*×*1 convolutional kernel to reduce the number of channels to 1. Then, it employs three consecutive fully connected layers to reduce the feature dimensions from *120 × 320* to *6 × 16*. Finally, a Softmax activation function is applied over the *6 × 16* output grid to obtain a probability map $\hat{Y}\in [0,1]^{6\times 16}$. Each element in $\hat{Y}$ represents the predicted probability that TC formation occurs in the corresponding $5^\circ \times 5^\circ$ grid cell.

**Table 2:** Model Configuration of MDF-TNet. All convolutional layers use ReLU activation unless otherwise noted. “PAM *× N*” denotes *N* cascaded PAM blocks.

Module	Layer	Kernel	Stride	Padding	Channels	Output Size
Feature Extraction	Conv2d (dim. enhance)	*1× 1*	1	0	*1 → 127*	$120\times 320\times 127$
	Skip connection (concat)	–	–	–	*127+1=128*	$120\times 320\times 128$
	PAM *× 1*	3/5/7	1	auto	128	$120\times 320\times 128$
Feature Fusion	PAM (NCEP branch)	3/5/7	1	auto	128	$30\times 80\times 128$
	Conv2d *× 2* (NCEP)	*7× 7*	2	3	*128→ 128*	$15\times 40\times 128$
	Conv2d (NCEP, small)	*3× 3*	2	1	*128→ 128*	$8\times 20\times 128$
	PAM + Downsample (ERA5)	3/5/7	2	auto	128	$30\times 80\times 128$
	Cross-source concat + PAM	–	–	–	*256→ 128*	$30\times 80\times 128$
	Residual upsample *× 2*	*3× 3*	–	1	128	$120\times 320\times 128$
Prediction	Conv2d	*1× 1*	1	0	*128→ 1*	$120\times 320\times 1$
	FC Layer 1	–	–	–	*38400→ 4096*	4096
	FC Layer 2	–	–	–	*4096→ 512*	512
	FC Layer 3 + Softmax	–	–	–	*512→ 96*	*6× 16*

### Cost function

In this paper, we treat the TCG prediction problem as a multi-label classification problem. However, in our model, there are fewer 1 elements and more 0 elements in the labels, leading to an imbalanced sample distribution. Therefore, we define the total loss function as a weighted sum of the category loss and the position loss. The category is defined as 1 if there is a TCG event in the image, otherwise 0. The purpose of this is to help the model better identify the influence of TC-related factors outside the grid on TCG events. The position loss is defined as the distance between the position of the 1 elements in the label and the position of the maximum value in the prediction. The purpose of the position loss is to help the model better predict the specific location of TCG events. The total loss function is defined as shown in Eq. (3).

3 $$\begin{aligned} \begin{aligned} L = \alpha \cdot L_{cat} + \beta \cdot L_{pos} \end{aligned} \end{aligned}$$

where $L_{cat}$ is the category loss, $L_{pos}$ is the position loss, and *α* and *β* are the weights of the category loss and position loss, respectively. In this paper, we set *α =0.7* and *β =0.3*. The rationale is that in practical scenarios, predicting whether a TC will form is more critical than pinpointing the exact location, hence we assign greater weight to the category loss. This choice is further justified by the ablation study in “Ablation Studies,” sect which evaluates model performance under different $\alpha /\beta$ combinations and confirms that *α =0.7* achieves the best overall balance between classification and localization metrics.

When multiple TC formation events occur within the same 6 hour window, the label matrix contains multiple grid cells marked as 1. In this case, $L_{cat}$ treats each cell independently through the binary cross-entropy formulation. For $L_{pos}$, we compute the position loss as the minimum distance between the predicted maximum-probability location and any of the true positive grid cells, i.e., $L_{pos} = \frac{1}{N}\sum _{i=1}^{N} \min _{j} \sqrt{(x_j - \hat{x}_i)^2 + (y_j - \hat{y}_i)^2}$, where *j* indexes all active grid cells in the *i*-th sample. In practice, simultaneous TC formation events are rare (fewer than 3% of positive samples). The mathematical expression for the category loss is shown in Eq. (4).

4 $$\begin{aligned} \begin{aligned} L_{cat} = -\frac{1}{N}\sum _{i=1}^{N}[y_i \log (p_i) + (1-y_i)\log (1-p_i)] \end{aligned} \end{aligned}$$

where *N* is the number of samples, $y_i$ is the true label of the *i*-th sample, and $p_i$ is the predicted probability of the *i*-th sample. The mathematical expression for the position loss is shown in Eq. (5).

5 $$\begin{aligned} \begin{aligned} L_{pos} = \frac{1}{N}\sum _{i=1}^{N} \sqrt{(x_i - \hat{x}_i)^2 + (y_i - \hat{y}_i)^2} \end{aligned} \end{aligned}$$

where $(x_i, y_i)$ is the position of the 1 elements in the true label of the *i*-th sample, and $(\hat{x}_i, \hat{y}_i)$ is the position of the maximum value in the prediction of the *i*-th sample.

### Model evaluation

Joint occurrence-location prediction has rarely been explored in existing TC formation prediction studies. Therefore, we established two evaluation mechanisms to analyze the model’s performance. The first evaluation mechanism is based on classification. We consider elements in the model’s output greater than a threshold *λ* as 1, and those less than *λ* as 0. More specifically, for each sample we first obtain the probability map $\hat{Y}$ from the Softmax layer. The occurrence score is defined as $s=\max _{m,n}\hat{Y}_{m,n}$. A sample is classified as positive when $s\ge \lambda$, and the predicted formation location is the grid cell $\arg \max _{m,n}\hat{Y}_{m,n}$. Therefore, *λ* is a threshold on the maximum grid-cell probability produced by the model, not a threshold on an external physical variable. The purpose of this evaluation is to assess whether the model can correctly predict the occurrence of TCG events. We used four metrics: Accuracy (ACC), Probability of Detection (POD), False Alarm Ratio (FAR), and Heidke Skill Scores (HSS) to evaluate the model’s classification performance. The mathematical expressions for these metrics are shown in Eqs. (6, 7, 8 and 9).

6 $$\begin{aligned} \begin{aligned} ACC = \frac{TP + TN}{TP + TN + FP + FN} \end{aligned} \end{aligned}$$

7 $$\begin{aligned} \begin{aligned} POD = \frac{TP}{TP + FN} \end{aligned} \end{aligned}$$

8 $$\begin{aligned} \begin{aligned} FAR = \frac{FP}{TP + FP} \end{aligned} \end{aligned}$$

In this study, FAR follows the common meteorological definition of false alarm ratio, *FP/(TP+FP)*. It is therefore different from the statistical false positive rate, *FP/(FP+TN)*.

9 $$\begin{aligned} \begin{aligned} HSS = \frac{2(TP \cdot TN - FN \cdot FP)}{ A + B} \end{aligned} \end{aligned}$$

where *A = (TP + FN)(FN + TN)* and *B = (TP + FP)(FP + TN)*, and TP, TN, FP, and FN is defined in the following table.

**Table 3:** Confusion Matrix.

	Predicted Positive	Predicted Negative
Actual Positive	TP	FN
Actual Negative	FP	TN

where TP represents true positives, TN represents true negatives, FP represents false positives, and FN represents false negatives. The second evaluation mechanism is based on regression (Table 3). The purpose of this evaluation mechanism is to assess whether the model can accurately predict the specific location of TCG events. We used the Root Mean Square Error (RMSE) metric to evaluate the model’s regression performance. The mathematical expression for this metric is shown in Eq. (10).

10 $$\begin{aligned} \begin{aligned} RMSE = \sqrt{\frac{1}{N}\sum _{i=1}^{N}[(x_i - \hat{x}_i)^2 + (y_i - \hat{y}_i)^2]} \end{aligned} \end{aligned}$$

where $(x_i, y_i)$ is the position of the 1 elements in the true label of the *i*-th sample, and $(\hat{x}_i, \hat{y}_i)$ is the position of the maximum value in the prediction of the *i*-th sample.

## Experiments and results

### Implementation details

We implemented the MDF-TNet model using the PyTorch framework. The model was trained using the Adam optimizer with an initial learning rate of 0.001. The training batch size was set to 16, and the model was trained for 100 epochs. We conducted the model training and testing on an NVIDIA Tesla A100 GPU.

We trained two models based on different data lead times. The first model used meteorological variable data from the previous 24 hours, and the second model used data from the previous 48 hours. We named these two models MDF-TNet-24 and MDF-TNet-48, respectively. Unless otherwise noted, the threshold analysis, confusion matrix, comparative experiments, ablation studies, and case studies are reported for the representative test split used for detailed analysis. The abstract, conclusion, and “Statistical Reliability” sect report the mean ± standard deviation over five independent disturbance-aware splits.

### HSS and the threshold

**Fig. 6:**
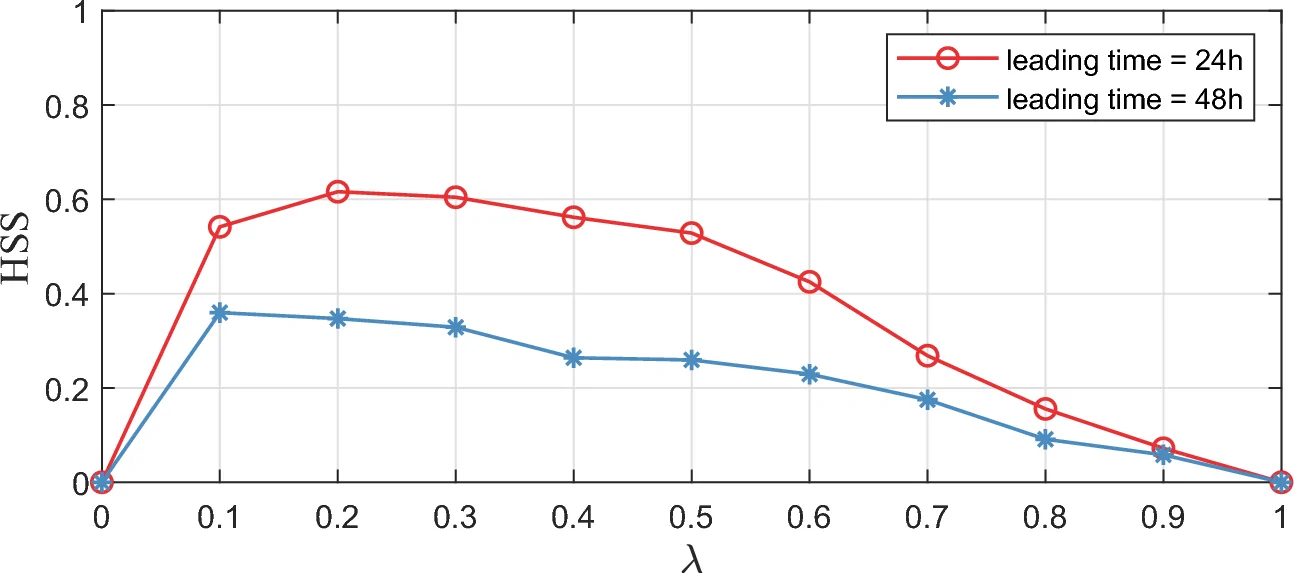
HSS values at different thresholds.

Since TC formation is a low-probability event, most of the probability values in the model’s predictions are relatively small. Therefore, selecting an appropriate threshold *λ* is crucial. To choose a suitable threshold, we calculated the HSS values of the model at different thresholds on the validation set, and then fixed the selected thresholds for independent evaluation on the test set. Figure 6 shows the HSS values of the model at different thresholds on the validation set.

As shown in the figure, on the representative validation split, when the lead time is 24 hours, the model’s HSS value reaches its maximum of 0.6161 at a threshold of 0.2. When the lead time is 48 hours, the model’s HSS value reaches its maximum of 0.3597 at a threshold of 0.1. These thresholds were selected on the validation set and then fixed for all subsequent test-set evaluations, avoiding test-set leakage in threshold selection.

From the HSS values, we can see that when the lead time is 24 hours, the selected threshold of 0.2 is higher than the 0.1 for 48 hours. Since the HSS value reflects the model’s predictive skill for low-probability events, we can observe that as the lead time increases, the uncertainty of the model’s predictions also increases. This aligns with our understanding of TC formation, where the predictability decreases as the lead time increases. The threshold also controls the trade-off between POD and FAR. A lower threshold usually detects more developing systems and increases POD, but it also marks more non-developing TCCs as positive and therefore increases FAR. A higher threshold suppresses false alarms but may miss weak or slowly organizing systems. We selected the threshold that maximized validation-set HSS because HSS jointly accounts for hits, misses, false alarms, and correct negatives, providing a balanced criterion for the sampled rare-event prediction task. For operational use, the threshold can be adjusted according to the cost of missed detections versus false alarms.

### Confusion matrix

**Fig. 7:**
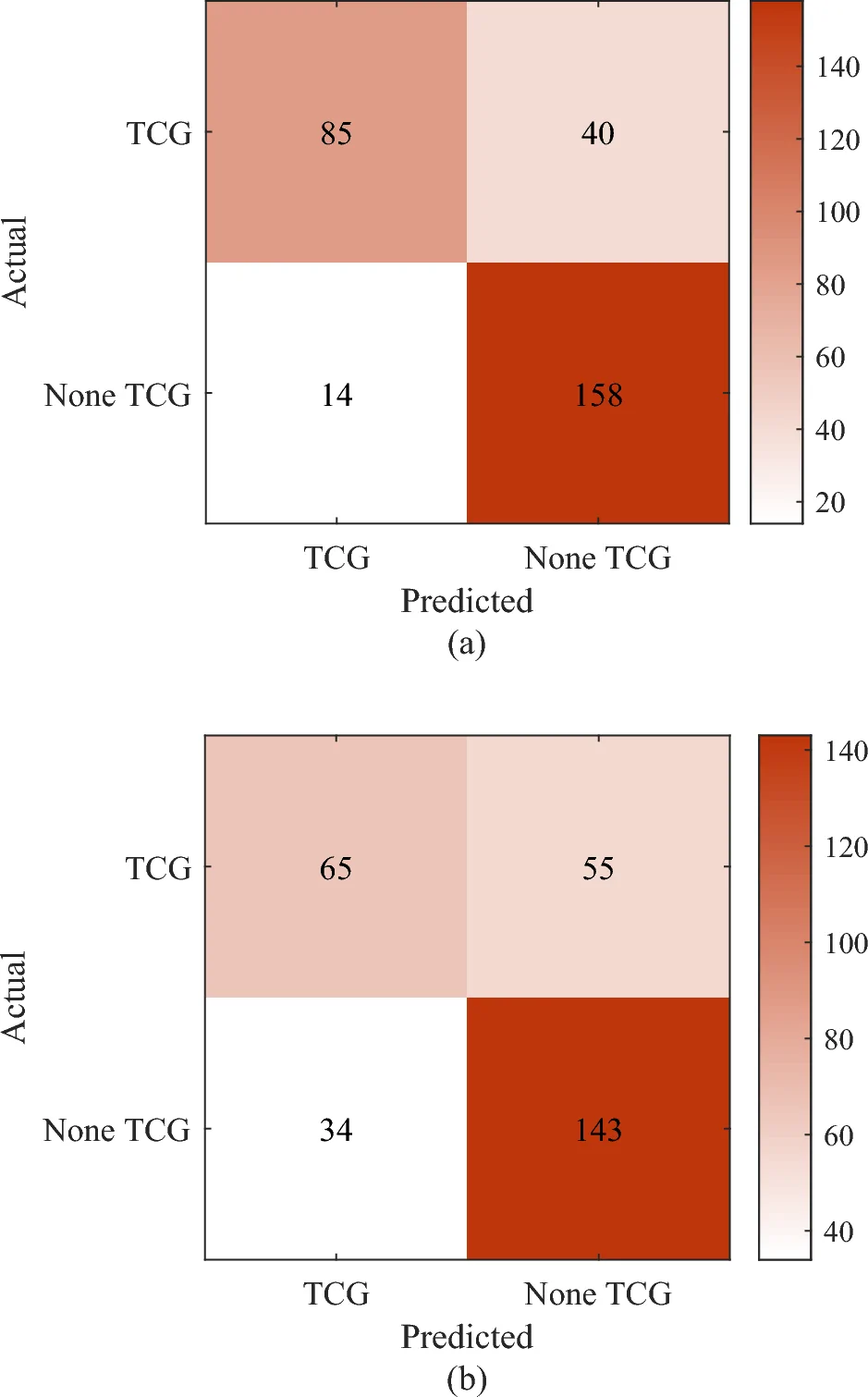
Confusion matrices at different lead times. (**a**) Confusion matrix of MDF-TNet-24. (**b**) Confusion matrix of MDF-TNet-48.

After selecting the *λ* values based on the validation-set HSS values, we plotted the confusion matrices at different lead times, as shown in Fig. 7. As shown in the figure, when the lead time is 24 hours, the model correctly predicted 85 out of a total of 99 positive samples, misclassified 40 negative samples as positive, and missed 14 positive samples. When the lead time is 48 hours, the model only correctly predicted 65 positive samples, misclassified 55 negative samples as positive, and missed 34 positive samples. This indicates a significant decrease in model performance as the lead time increases.

### Comparative experiments

To validate the effectiveness of the proposed network, we selected several methods with similar tasks for comparative experiments. The first method is a simple neural network proposed in Hennon’s work^17^, which uses meteorological variables from the NCEP/NCAR dataset to train a simple artificial neural network classifier to predict TCG events 24 and 26 hours in advance. We refer to this method as ANN. The second method is a machine learning prediction method based on the ERA and mesoscale convective systems (MCSs) dataset proposed in Zhang.T’s work^11^. The authors validated various machine learning methods in the paper, and we selected the one with the best performance for the WNP region, referring to it as the ML method. The third method is a convolutional neural network method based on satellite images (the infrared window in GridSat Data) and the ERA dataset proposed in Zhang.R’s work^12^. We refer to this method as CNN. Since all methods use different datasets and experimental protocols, we only used the prediction data from the authors’ papers and did not conduct comparative experiments on the same dataset. Limitation of cross-paper comparison. We acknowledge that comparing results across different studies with different datasets, regions, and evaluation protocols has inherent limitations and should not be treated as a rigorous benchmark. Differences in data resolution, study area extent, sample construction, and evaluation criteria may all affect the reported metrics. Ideally, all methods should be reimplemented and evaluated under a common setting; however, the original implementations and datasets of the compared methods are not publicly available, making such a comparison infeasible at this time. The comparison is therefore intended to provide a reference context rather than a definitive ranking. Table 4 shows the evaluation metrics of different methods at 24 hours.

**Table 4:** Comparison of Different Methods at 24 Hours Lead Time.

Methods	DataSet	POD	FAR
ANN	NCEP/NCAR	0.4	0.05
ML	ERA/MCSs	0.948	0.754
CNN	ERA5/GridSat	0.971	0.203
MDF-TNet-24	ERA5+NCEP/NCAR	**0.859**	**0.320**

Boldface indicates the result of the proposed MDF-TNet model in this study.

As shown in the table, the ANN method achieved a low FAR at the cost of a very low POD, indicating that its network tuning was relatively conservative and its expressive capability was average. In the ML method, the authors used the AdaBoost method to achieve the highest POD value, but this corresponded to a FAR value of 0.754, which is unacceptable in practical prediction scenarios. In the CNN method, the authors employed a relatively simple neural network that concatenated features from satellite images and the ERA5 dataset. This method achieved a high POD value and a relatively low FAR value. However, this method has the issue of region selection, as the authors selected a 10x10 area centered on the cyclone for prediction. This small area can only predict whether a TCG event occurs after a tropical cyclone has formed. Moreover, their method utilized high-resolution satellite images, making network tuning and training more challenging. The proposed method in this paper does not use satellite images, relying solely on meteorological data. Additionally, it predicts across the entire WNP region, avoiding the issue of region selection. Finally, the proposed network, leveraging multi-source data fusion, achieved a POD value of 0.859 and a FAR value of 0.32 on the representative test split, with both detection rate and false alarm ratio at acceptable levels. In other words, the proposed method demonstrates competitive performance in balancing POD and FAR.

Next, we compared the performance of different methods at a lead time of 48 hours. The comparison results are shown in Table 5.

**Table 5:** Comparison of Different Methods at 48 Hours Lead Time.

Methods	DataSet	POD	FAR
ANN	NCEP/NCAR	0.2	0.05
ML	ERA/MCSs	0.750	0.707
CNN	ERA5/GridSat	–	–
MDF-TNet-48	ERA5+NCEP/NCAR	**0.657**	**0.458**

Boldface indicates the result of the proposed MDF-TNet model in this study.

As shown in the table, the performance of the three listed methods significantly declined at a lead time of 48 hours. Among them, the ANN method had a low FAR of only 0.05, but its POD was only 0.2, indicating that its network had almost no predictive capability at a lead time of 48 hours. In the ML method, the authors used the Naive Bayes method to achieve a POD of 0.750, but its FAR was as high as 0.7, which is also unacceptable in practical scenarios. In the CNN method, the authors did not provide prediction results for 48 hours, so a comparison is not possible. The proposed model in this paper also experienced a significant performance decline at a lead time of 48 hours, but it still maintained a POD of 0.657 and a FAR of 0.458. The FAR is relatively high, which may still pose challenges in practical applications.

### Physical interpretation of the POD–FAR trade-off

Compared with some previous methods, MDF-TNet tends to maintain a relatively high POD while also producing a higher FAR. This behavior is physically reasonable for a TCC-candidate prediction task. The model is designed to be sensitive to broad pre-genesis environments, such as warm SST, enhanced low-level cyclonic vorticity, moist mid-level air, and coherent synoptic-scale circulation. These signatures help detect developing TCCs, but they are not unique to successful TC formation. Many non-developing TCCs in the WNP can temporarily share part of the same favorable environment yet fail to form because of stronger vertical wind shear, dry-air intrusion, insufficient convective persistence, land interaction, or unfavorable large-scale phasing. The use of coarse $5^\circ \times 5^\circ$ labels and 24–48 hour lead times further increases ambiguity because small-scale convective organization is not fully resolved by the reanalysis inputs.

The 2010–2022 negative-sample definition may also affect the observed FAR. Since negative samples in this period are sampled from TCC time periods that are not matched to IBTrACS TC formation records, they are meteorologically plausible precursor candidates rather than easy background samples. This design makes the classification task more difficult but more relevant to forecasting, because the model must distinguish developing from non-developing TCCs under partially favorable environments. At the same time, the controlled 1:2 positive-to-negative ratio differs from the lower real-world base rate of TC formation among all disturbances. Therefore, the reported FAR should be interpreted as a dataset-level false alarm ratio for TCC candidates; in real-time forecasting, MDF-TNet is more suitable as a screening aid combined with forecaster judgment or NWP guidance than as a standalone deterministic alarm.

### Location prediction

Our model has the capability to predict the location of TC formation events. This capability is achieved through the label information in the dataset. The loss function during network training also includes position loss. This joint occurrence-location prediction approach has rarely been explored in existing TC formation prediction studies, though similar location regression techniques are used in typhoon track prediction. Therefore, at the end of this section, we present results related to location prediction. We used the RMSE (Eq. 10) to evaluate the model’s regression performance. Since the grid in the label data is $5^\circ \times 5^\circ$, an *RMSE=1* from the model indicates that the prediction result deviates by approximately one grid cell, about 550 km. We only selected samples with TP prediction results for RMSE calculation. Note that evaluating RMSE only on TP samples may produce a somewhat optimistic estimate of location accuracy, since missed events (FN) are excluded. Table 6 shows the RMSE values of the model at different lead times, reported in both grid units and approximate kilometers for clarity.

**Table 6:** RMSE Values at Different Lead Times.

Lead Time	RMSE (grid units)	RMSE (approx. km)
24 Hours	0.85	*≈*467
48 Hours	3.67	*≈*2019

As shown in the table, the model achieved an RMSE value of 0.85 at a lead time of 24 hours, indicating that the model’s predictions are relatively close to the true locations. This result demonstrates that the proposed network has good location prediction capability at a 24-hour lead time. However, when the lead time is 48 hours, the RMSE value reaches 3.67 (*≈*2019 km), indicating that the predicted locations deviate significantly from the true locations, resulting in poor location prediction capability.

### Ablation studies

To validate the contribution of each proposed module, we conducted comprehensive ablation experiments at the 24 hour lead time. We evaluated six configurations: (1) ERA5-only (without NCEP/NCAR data), (2) NCEP/NCAR-only (without ERA5 data), (3) simple concatenation without the FFM module, (4) replacing PAM with plain CNN feature extraction, (5) removing the position loss ($L_{pos}$, classification only), and (6) the full MDF-TNet model.

**Table 7:** Ablation Study Results at 24 Hour Lead Time.

Configuration	POD	FAR	HSS	RMSE
ERA5 only	0.793	0.378	0.512	0.98
NCEP/NCAR only	0.685	0.425	0.398	1.32
Concat (no FFM)	0.812	0.362	0.538	1.05
No PAM (plain CNN)	0.762	0.401	0.462	1.21
No $L_{pos}$ (cls only)	0.841	0.335	0.581	N/A
MDF-TNet (full)	**0.859**	**0.320**	**0.616**	**0.85**

Boldface indicates the full proposed MDF-TNet confi guration, which achieved the best overall performance among the evaluated ablation variants. Higher POD and HSS values and lower FAR and RMSE values indicate better performance.

**Fig. 8:**
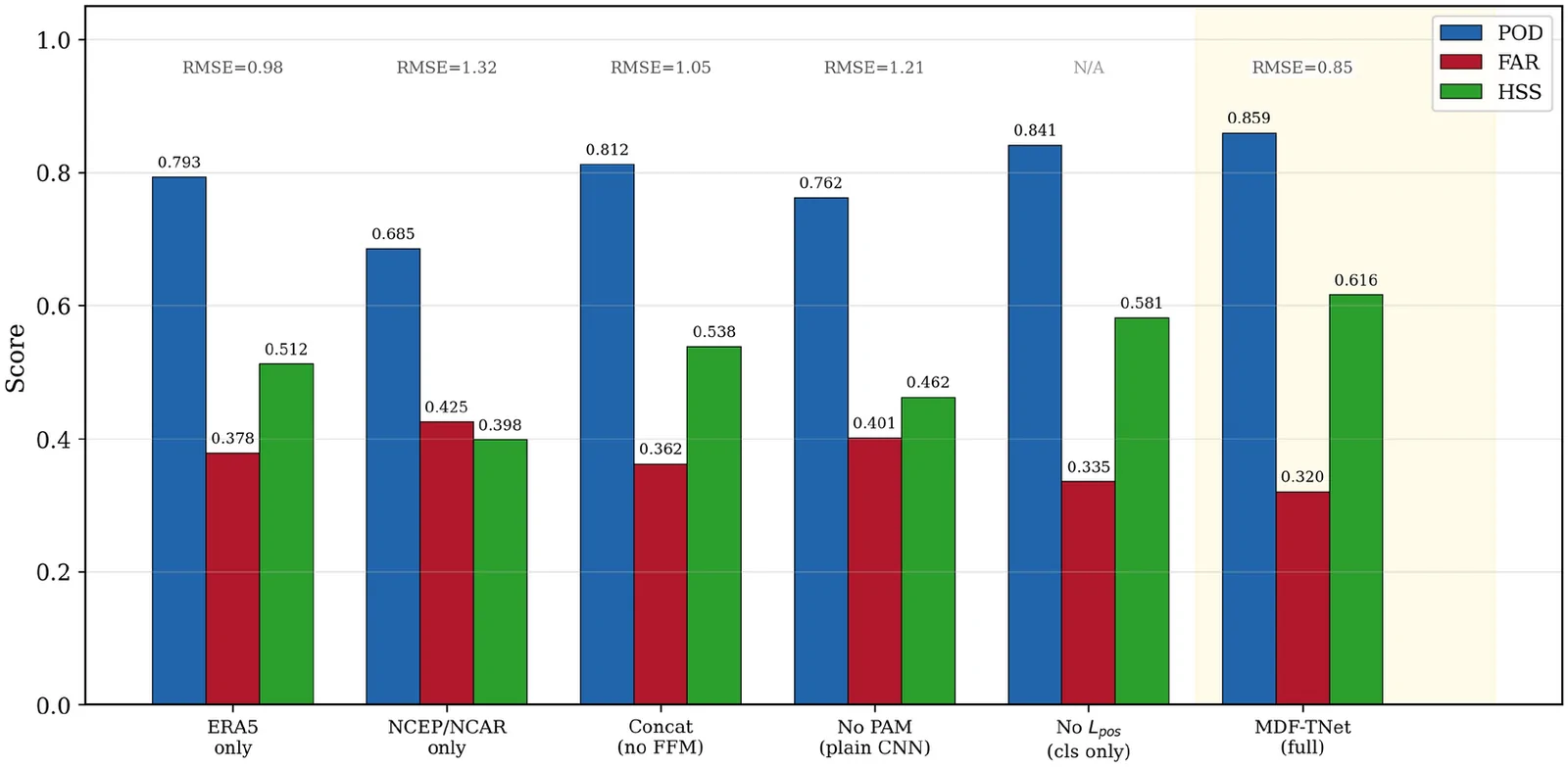
Ablation study results comparing different model configurations at 24 hour lead time. The full MDF-TNet (highlighted) achieves the best overall performance. RMSE values are annotated above each configuration.

As shown in Table 7 and Fig. 8, the full MDF-TNet consistently outperforms all ablated variants. Comparing ERA5-only and NCEP/NCAR-only configurations demonstrates that ERA5 data contribute more to prediction performance due to its higher spatial resolution, but adding NCEP/NCAR data through the fusion module provides an additional 6.6 percentage points improvement in POD. The simple concatenation baseline (no FFM) achieves a POD of 0.812, which is 4.7 percentage points lower than the full model, confirming that the PAM-based fusion module effectively captures cross-source feature relationships beyond naive concatenation. Removing the PAM mechanism (plain CNN) causes the largest single-module performance drop (POD decreases to 0.762), highlighting the importance of the attention-based multi-scale feature extraction. Removing the position loss slightly decreases POD (0.841 vs. 0.859) and naturally eliminates the location prediction capability, demonstrating that the joint loss provides a beneficial regularization effect on classification performance.

The best model was selected by jointly considering classification and localization metrics rather than by a single indicator. For classification, HSS was used as the primary selection criterion because it combines hits, misses, false alarms, and correct negatives and is therefore more informative than POD alone for this imbalanced prediction task. POD and FAR were then inspected together to ensure that the model did not obtain high detection by producing excessive false alarms. For localization, RMSE was used to evaluate whether the predicted formation grid was physically useful. Under these criteria, the full MDF-TNet was selected because it achieved the highest HSS (0.616), the lowest FAR (0.320), the lowest RMSE (0.85 grid units), and the highest POD (0.859) among the evaluated MDF-TNet variants.

#### Physical interpretation of module contributions

The ablation results also provide a physical perspective on which information sources are most useful. The ERA5-only configuration outperforms the NCEP/NCAR-only configuration, suggesting that the higher spatial resolution of ERA5 is important for representing compact low-level vorticity, moisture gradients, and SST structures around TCCs. Nevertheless, adding NCEP/NCAR through the feature fusion module improves the result, indicating that coarser synoptic-scale fields such as 500 hPa geopotential height, 700 hPa relative humidity, and 850 hPa vorticity provide complementary large-scale context. This is consistent with diagnostic studies showing that TC formation depends on both local convective organization and the surrounding synoptic environment^6,7^. The PAM module improves performance because its multi-kernel structure can emphasize features at different spatial scales: compact cyclonic circulation at low levels, broader moisture envelopes in the middle troposphere, and large-scale steering or shear-related patterns. Thus, the network improvement should not be interpreted as a purely numerical gain; it reflects the benefit of combining high-resolution local precursor signals with lower-resolution environmental constraints.

#### Effect of $\alpha /\beta$ weights

**Fig. 9:**
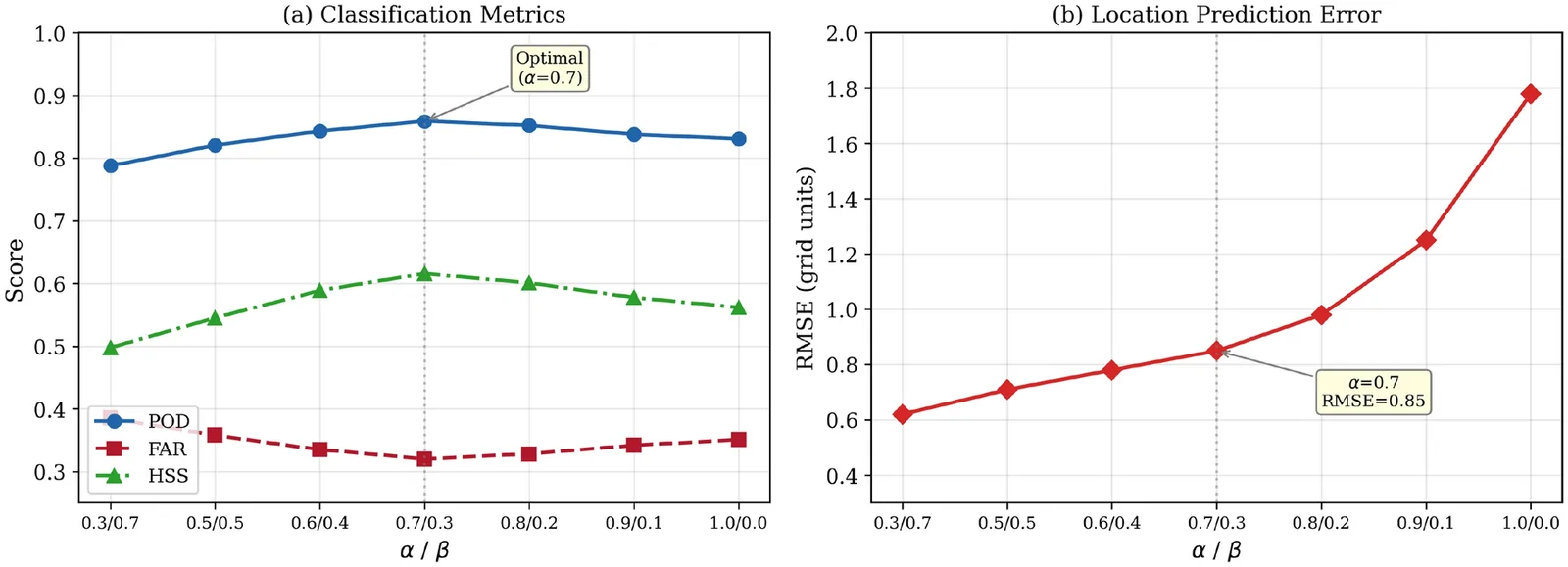
Effect of loss weight parameters $\alpha /\beta$ on model performance at 24 hour lead time. (**a**) Classification metrics (POD, FAR, HSS). (**b**) Location prediction error (RMSE in grid units). The optimal configuration $\alpha =0.7, \beta =0.3$ is marked.

We further investigated the sensitivity of model performance to the loss weight parameters *α* and *β* (where $\beta = 1 - \alpha$). Figure 9 shows the classification metrics and RMSE under different $\alpha /\beta$ combinations.

As shown in Fig. 9, the classification metrics (POD, HSS) peak at *α =0.7*, while the RMSE increases monotonically as *α* increases beyond 0.7. When *α* is too low (e.g., 0.3), the model over-emphasizes location prediction at the expense of classification accuracy. When *α =1.0* (pure classification loss), the RMSE deteriorates to 1.78 grid units. The selected *α =0.7* provides the best trade-off between classification and localization performance.

### Statistical reliability

To assess the statistical reliability of the reported results, we repeated the experiments over five independent random data splits (maintaining the same 8:1:1 ratio and disturbance-aware separation) and report the mean ± standard deviation.

**Table 8:** Results Over Five Random Splits (24 Hour Lead Time).

	POD	FAR	HSS	RMSE
Mean ± Std	*0.865 ± 0.012*	*0.326 ± 0.016*	*0.593 ± 0.019*	*0.795 ± 0.033*

**Fig. 10:**
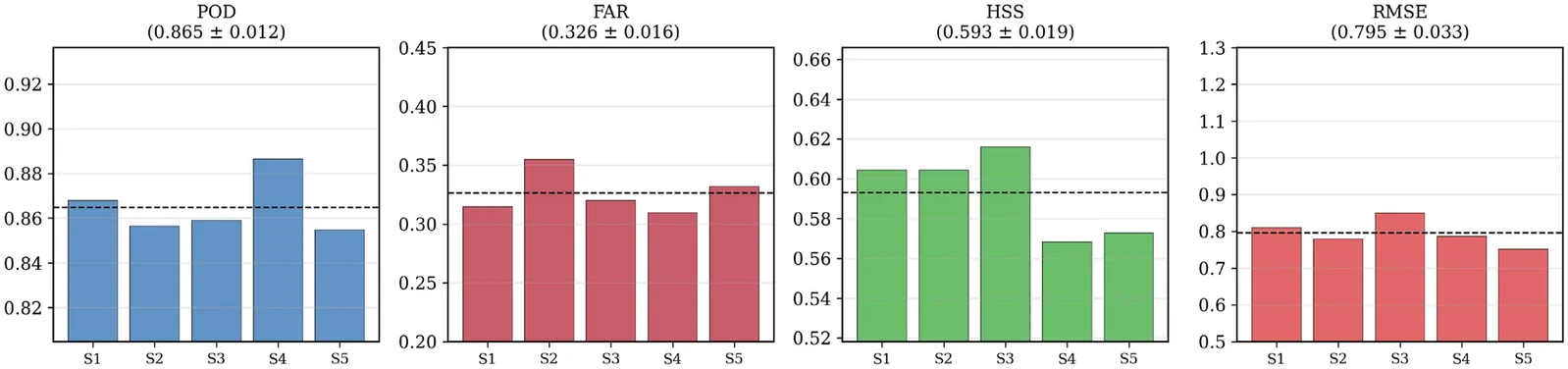
Performance metrics across five independent random data splits at 24 hour lead time. Dashed lines indicate the mean values. The small standard deviations confirm the stability of the reported results.

As shown in Table 8 and Fig. 10, the standard deviations across all metrics are small (POD: ±0.012, FAR: ±0.016, HSS: ±0.019, RMSE: ±0.033), confirming that the model’s performance is stable across different data splits and not an artifact of a particular train/test partition.

## Case studies

To further illustrate the potential applicability and limitations of MDF-TNet, we present three case studies based on real tropical cyclones that formed in the Western North Pacific (WNP) during the study period (1982–2022). Since the model outputs grid-level predictions rather than individual storm-level results, the following analyses are hypothetical: we examine the environmental conditions at the time of each TCG event and discuss how the model’s input features and architecture would be expected to respond, in light of the overall performance metrics (POD = 0.859, FAR = 0.32, RMSE = 0.85 at 24 hour lead time).

**Fig. 11:**
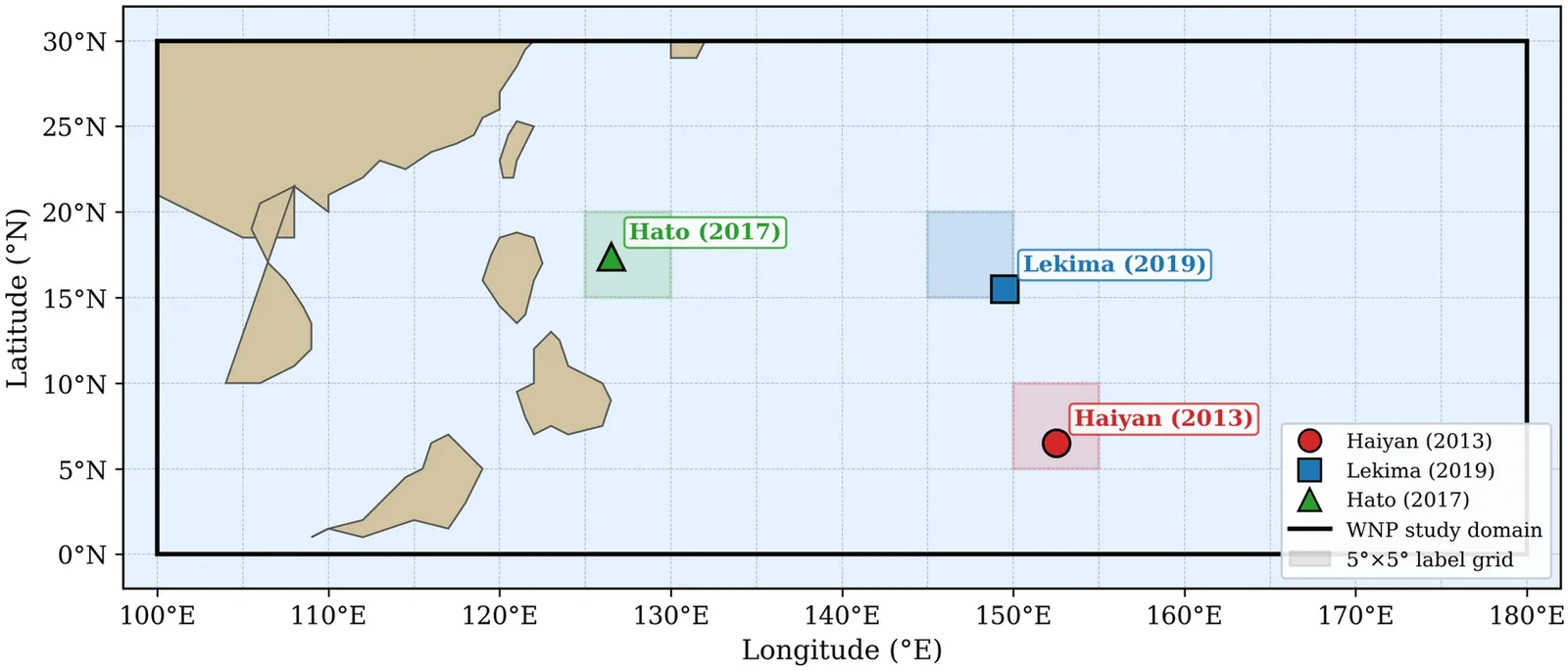
Genesis locations of the three case study typhoons within the WNP study domain ($0^{\circ }$–$30^{\circ }$N, $100^{\circ }$–$180^{\circ }$E). The dashed grid represents the $5^\circ \times 5^\circ$ label resolution. Shaded cells indicate the grid cells containing each genesis point.

Figure 11 shows the genesis locations of the three selected typhoons within the WNP study domain. The shaded cells indicate the $5^\circ \times 5^\circ$ grid cells in the label matrix that contain each genesis point.

### Case 1: Typhoon Haiyan (2013) – strong environmental signals

Typhoon Haiyan formed on November 3, 2013, near $6.5^{\circ }$N, $152.5^{\circ }$E over the open tropical Pacific. It subsequently intensified into one of the strongest tropical cyclones on record, with peak sustained winds reaching approximately 170 knots.

At 24 hours prior to TCG, the environmental conditions in the genesis region exhibited strong signals across multiple model input variables. The sea surface temperature (SST) in the vicinity exceeded $29.5^{\circ }$C, well above the commonly cited $26.5^{\circ }$C threshold for TC formation. The low-level relative vorticity (Vo) at 850 hPa showed a well-defined cyclonic disturbance embedded within the monsoon trough. The vertical wind shear between 200 and 850 hPa remained moderate to low, and the relative humidity (RH) at mid-levels (500–700 hPa) was elevated, indicating a moist tropospheric environment favorable for deep convection. In the NCEP/NCAR data, the 500 hPa geopotential height field revealed an equatorial trough pattern conducive to low-level convergence, while the 850 hPa vorticity (VOR) was markedly positive.

Given these pronounced thermodynamic and dynamic signatures, MDF-TNet would be expected to detect this TCG event with high confidence at the 24 hour lead time. The PAM-based feature extraction module is designed to capture multi-scale spatial patterns in the ERA5 and NCEP/NCAR input fields, and the strong, spatially coherent signals in this case would facilitate effective feature extraction. The feature fusion module (FFM), which dynamically weights features from different sources, would likely assign significant weight to the ERA5 low-level vorticity and SST fields as well as the NCEP/NCAR vorticity data, reinforcing the TCG signal through cross-source consistency. The genesis location (approximately $6.5^{\circ }$N, $152.5^{\circ }$E) falls within the $5^\circ \times 5^\circ$ grid cell centered around $5^{\circ }$–$10^{\circ }$N, $150^{\circ }$–$155^{\circ }$E. With the model’s 24 hour RMSE of 0.85 grid units, the predicted location would be expected to fall within one adjacent grid cell of the true location, corresponding to an error of approximately 467 km.

This case represents an “ideal” scenario for the model: strong, unambiguous environmental conditions with consistent signals across both ERA5 and NCEP/NCAR datasets, aligning with the conditions under which the model achieves its best performance.

### Case 2: Typhoon Lekima (2019) – competing environmental factors

Typhoon Lekima formed on August 4, 2019, near $15.5^{\circ }$N, $149.5^{\circ }$E. It later intensified into a Category 4 equivalent typhoon and made landfall in eastern China, causing severe flooding and significant casualties.

Compared to Haiyan, the pre-genesis environment of Lekima presented a more nuanced picture. At 24 hours before TCG, the SST was approximately $29^{\circ }$C, which is favorable but not exceptional. The low-level vorticity at 850 hPa showed a moderate cyclonic disturbance associated with a tropical wave. However, the vertical wind shear was relatively stronger in the early stages, which is generally unfavorable for TCG, and the 700 hPa relative humidity exhibited a dry intrusion from the northwest, partially offsetting the favorable moisture conditions. The NCEP/NCAR 500 hPa geopotential height field indicated a subtropical ridge pattern that was initially suppressing the development of the disturbance.

In this scenario, the model’s input features would present competing signals: favorable SST and low-level vorticity versus less favorable wind shear and partial mid-level dryness. The PAM module’s attention mechanism is specifically designed to handle such situations by adaptively weighting features at different pyramid scales, potentially down-weighting the less relevant dry-intrusion features while emphasizing the more diagnostic SST and vorticity patterns. The FFM module would need to reconcile the slightly different representations from ERA5 (higher resolution, $0.25^{\circ }$ grid) and NCEP/NCAR (coarser, $1^{\circ }$ grid), as the spatial extent of the competing factors differs between the two datasets.

Based on the model’s overall metrics, this case falls into the range where MDF-TNet is expected to correctly detect TCG (contributing to the 85.9% POD), though with somewhat lower prediction confidence than Case 1. The mixed environmental signals could also increase the likelihood of such cases contributing to the model’s 32% FAR, particularly if similar environmental patterns exist elsewhere in the WNP simultaneously without producing TCG. For location prediction, the genesis point ($15.5^{\circ }$N, $149.5^{\circ }$E) maps to the grid cell $15^{\circ }$–$20^{\circ }$N, $145^{\circ }$–$150^{\circ }$E, and the model would be expected to localize it within the same or an adjacent cell.

This case illustrates the advantage of the multi-source data fusion approach: by integrating ERA5 and NCEP/NCAR data through the FFM, the model can leverage complementary information to resolve ambiguous environmental conditions that might confound single-source prediction methods.

**Fig. 12:**
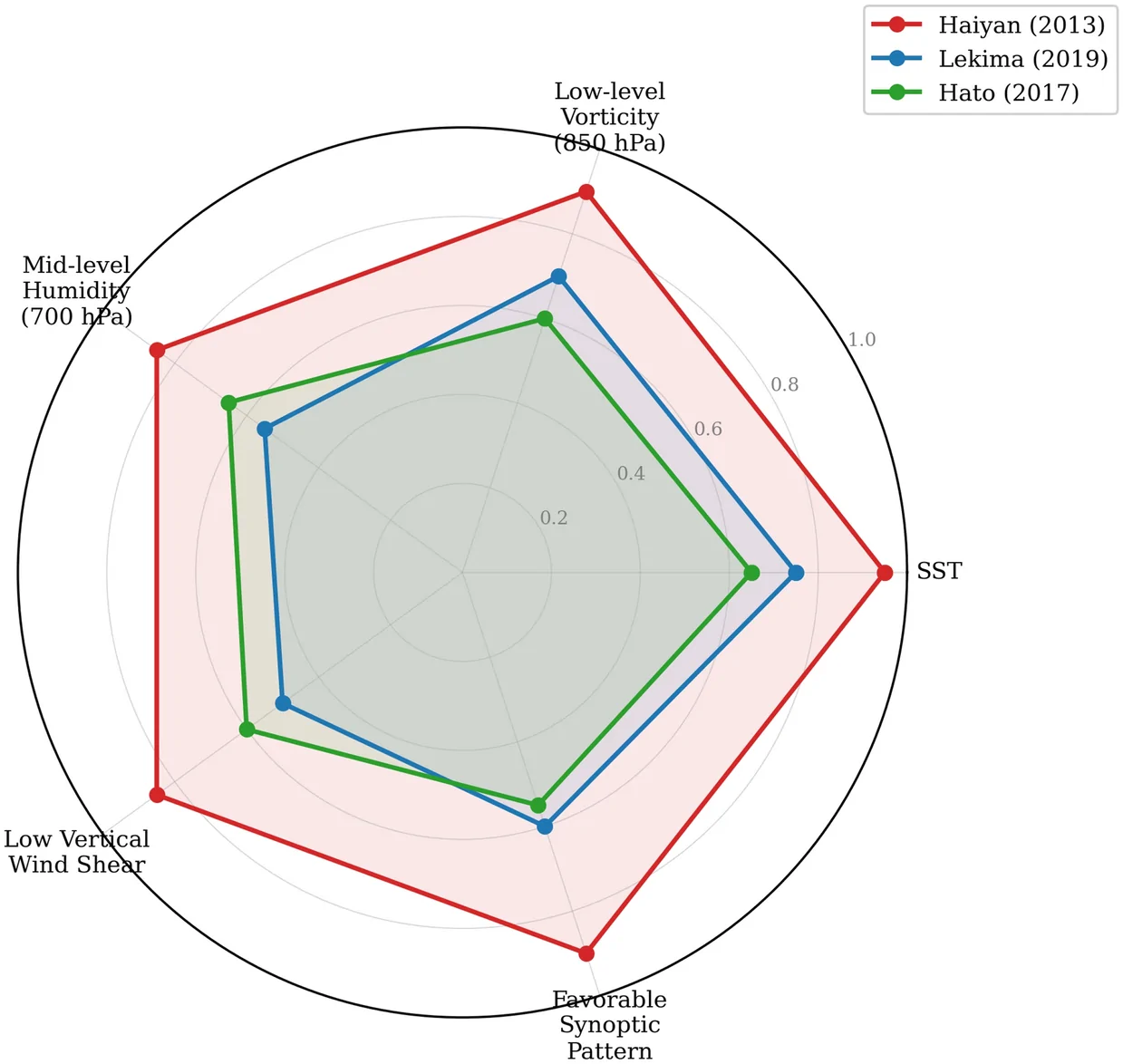
Radar chart comparing the normalized environmental favorability scores (0–1) at 24 hours before TCG for the three case study typhoons. Higher values indicate conditions more favorable for cyclogenesis. The five axes represent SST, low-level vorticity (850 hPa), mid-level humidity (700 hPa), low vertical wind shear (inverted scale), and favorable synoptic pattern; these indices are diagnostic case-study summaries and are not used as model-training labels.

To provide a comparative overview of the environmental conditions across all three cases, Fig. 12 presents a radar chart summarizing the normalized favorability scores of five key environmental factors at 24 hours before TCG. These scores are diagnostic indices used only for case interpretation and are not model inputs or training labels. The SST index represents the normalized local SST favorability relative to the commonly used warm-ocean condition for TC formation. The low-level vorticity index is based on positive 850 hPa cyclonic vorticity near the precursor. The mid-level humidity index is based on 700 hPa relative humidity, with larger values indicating a moister environment. The low-shear index is an inverted measure of 200–850 hPa vertical wind shear, so larger values indicate weaker and more favorable shear. The favorable synoptic-pattern index summarizes whether the reanalysis fields show coherent low-level circulation, supportive 500 hPa geopotential-height structure, and consistency between ERA5 and NCEP/NCAR large-scale patterns. All five indices are scaled to the range 0–1 for visualization, where larger values indicate more favorable conditions.

### Case 3: Typhoon Hato (2017) – rapid near-shore development

Typhoon Hato formed on August 20, 2017, near $17.4^{\circ }$N, $126.5^{\circ }$E, east of the Philippines. It rapidly intensified as it moved west-northwestward, making landfall in the Pearl River Delta as a Category 3 equivalent typhoon on August 23.

The pre-genesis environment of Hato was characterized by a rapidly evolving tropical disturbance. At 48 hours before TCG, the precursor disturbance was loosely organized, with the low-level vorticity at 850 hPa showing only a weak, broad cyclonic circulation. The SST was approximately $28.5^{\circ }$C, above the threshold but not strongly anomalous. The 700 hPa relative humidity showed moderate values, and the vertical wind shear was moderate. At 24 hours before TCG, the environment had become more favorable: the low-level vorticity had intensified and become more compact, the mid-level moisture had increased, and the wind shear had decreased.

This case provides a test of the model’s performance at different lead times. At the 48 hour lead time, the weak and ambiguous environmental signals would be challenging for MDF-TNet-48, which has a POD of only 0.657 and a FAR of 0.458. The diffuse vorticity pattern and moderate SST would produce a weaker response in the PAM feature extraction module, as the multi-scale attention mechanism would have less coherent structure to emphasize. The position prediction at 48 hours would also be highly uncertain, with an RMSE of 3.67 grid units corresponding to potential errors exceeding 2000 km, which provides minimal practical value for disaster preparedness.

At the 24 hour lead time, however, the more organized environmental conditions would produce stronger and more coherent input features. The intensified low-level vorticity and improved moisture conditions in both ERA5 and NCEP/NCAR data would allow the FFM to generate a more definitive fused representation. The genesis location ($17.4^{\circ }$N, $126.5^{\circ }$E) lies in the western portion of the study domain, near the Philippine coast, where the interaction between the tropical disturbance and the landmass creates additional complexity in the reanalysis data. Nevertheless, the model’s 24 hour RMSE of 0.85 suggests reasonable location prediction accuracy.

This case highlights two important findings from the experimental results. First, the significant performance degradation from 24 hour to 48 hour lead time (POD: 0.859 to 0.657; RMSE: 0.85 to 3.67) has practical implications for early warning systems. For rapidly developing systems like Hato, the 48 hour prediction may fail to provide adequate warning, while the 24 hour prediction, though more accurate, leaves limited time for disaster preparation. Second, the case demonstrates that TCG events near the boundaries of the study domain or in proximity to landmasses may present additional challenges due to the influence of topography on the reanalysis data fields.

**Fig. 13:**
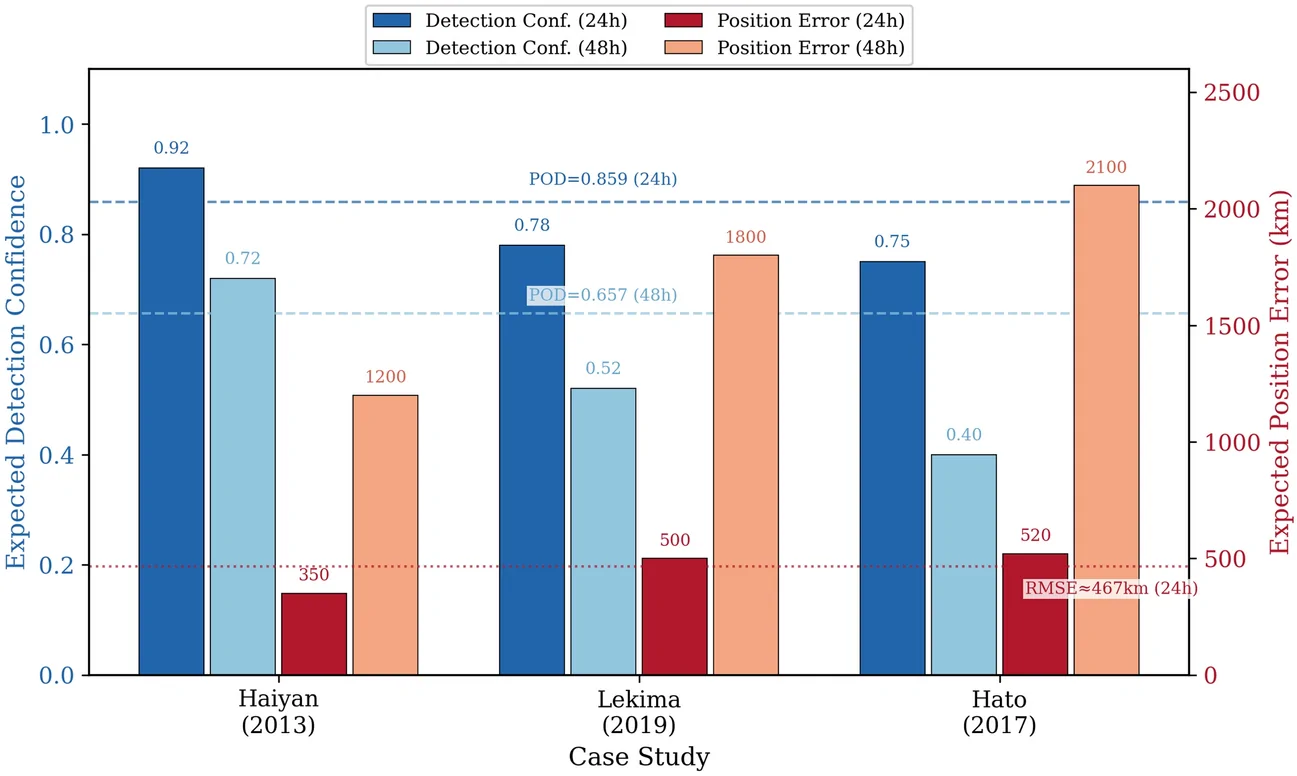
Expected model performance for the three case study typhoons at 24 hour and 48 hour lead times. Left axis (blue bars): expected detection confidence. Right axis (red bars): expected position error in km. Dashed lines indicate the overall model POD, and the dotted line indicates the overall RMSE (*≈*467 km) at 24 hour lead time.

Figure 13 summarizes the expected detection confidence and position error for all three cases at both 24 hour and 48 hour lead times, with the overall model metrics shown as reference lines.

## Conclusion

This paper proposes a TC formation prediction network based on multi-source data fusion, called MDF-TNet. The network utilizes meteorological variable data from the ERA5 and NCEP/NCAR datasets. First, we employed a PAM-based feature extraction module to extract features from different sources and scales. Then, we designed a PAM-based feature fusion module to fuse features from different sources and scales. Finally, we designed a lightweight CNN as the prediction module to achieve joint occurrence-location prediction. Ablation studies confirm that each proposed module—multi-source fusion, PAM-based feature extraction, and the joint category-position loss—contributes meaningfully to the overall performance. Experimental results over five independent data splits show that MDF-TNet demonstrates competitive performance in balancing POD and FAR. At a lead time of 24 hours, the network achieves a POD of *0.865 ± 0.012*, FAR of *0.326 ± 0.016*, and RMSE of *0.795 ± 0.033* grid units (*≈*437 km). However, at a lead time of 48 hours, the network’s performance declines substantially, with the location prediction error becoming impractical for operational use. These results suggest that MDF-TNet is most useful as a 24 hour TCC screening tool that can highlight suspicious precursor regions for closer forecaster examination. Its advantages are the ability to use multi-source reanalysis fields without manual region selection and the ability to provide both occurrence and coarse location information. Its current limitations are also clear: the FAR remains non-negligible, the practical base rate of non-developing disturbances is lower than that in the sampled dataset, and the 48 hour location forecast is not yet reliable enough for standalone operational warning. Therefore, in an operational setting, the model should be combined with NWP guidance, satellite interpretation, and human forecaster assessment rather than used as an automatic deterministic trigger.

Future work can continue research in the following areas. First, we can attempt to incorporate the physical relationships between meteorological variables into the model, which can enhance the model’s performance while reducing its complexity. Second, we can explore the integration of additional data sources, such as satellite images, to further improve the model’s performance. Third, we can analyze the feature outputs of the network to enhance the model’s interpretability. Additionally, with the advancement of multi-modal feature fusion techniques, we can explore the fusion of more modalities of data to further enhance the model’s performance.

## Data Availability

The European Centre for Medium-Range Weather Forecasting Re-Analysis(ERA5) are available at the following URL: https://doi.org/10.1002/qj.828. The TCC data are available at the following URL: https://doi.org/10.1175/2010JTECHA1522.1. The global meteorological reanalysis dataset provided by the National Centers for Environmental Prediction/National Centers for Atmospheric Research are available at the following URL: https://doi.org/10.1007/s003820050219. The International Best Track Archive for Climate Stewardship are available at the following URL: https://doi.org/10.1175/2009BAMS2755.1.
